# Abdominal computed tomography–assessed muscle quality and its prognostic value in patients with advanced chronic kidney disease initiating hemodialysis

**DOI:** 10.1371/journal.pone.0334929

**Published:** 2025-11-04

**Authors:** Suyeon Han, Hwajin Park, Yu Ah Hong, Yunkyeong Hwang, Yoon-Kyung Chang

**Affiliations:** 1 Division of Nephrology, Department of Internal Medicine, College of Medicine, The Catholic University of Korea, Seoul, Korea; 2 Daejeon St. Mary Hospital, The Catholic University of Korea, Daejeon, Korea; Shuguang Hospital, CHINA

## Abstract

**Background:**

Skeletal muscle density (SMD), assessed via L3 level computed tomography (CT), is a critical marker of muscle quality with remarkable prognostic value in various clinical settings. This study investigates prognostic values of the SMD of abdominal CT images and to verifies its relationship with other variables indicating sarcopenia in patients with advanced chronic kidney disease (CKD) patients.

**Methods:**

All 458 patients initiating hemodialysis for maintenance were enrolled in this retrospective study. The sex-specific cut-off values of the SMD and skeletal muscle index (SMI) of L3 level abdominal CT were obtained by drawing the receiver operating characteristic curve for mortality. Cut-offs for identifying low SMD and SMI groups were applied.

**Results:**

The mean age of all patients was 67 years, and 300 (65.6%) patients were male. A total of 204 (44.5%) patients died. In the fully adjusted Cox regression analysis, patients with low SMD had a higher risk of death than those with SMD above the reference cut-off (adjusted hazard ratio (adjHR): 1.805 95% confidence interval (CI): 1.263–2.579, *p*-value < 0.001). The continuous variable SMD had a prognostic value in the multivariate Cox proportional analysis (adjHR: 0.961, 95% CI: 0.942–0.980, *p*-value <0.001), while the continuous variable SMI did not have prognostic value after adjustment (adjHR: 1.002, 95% CI: 0.986–1.019, *p*-value = 0.778). The multivariate regression analysis for SMD with the clinical variable showed that male sex, younger age, lower body mass index (BMI), and higher albumin remained significantly and independently associated with higher SMD. SMD showed an inverse correlation with BMI in contrast to SMI.

**Conclusion:**

The low SMD assessed by abdominal CT is independently associated with all-cause mortality in patients with advanced CKD initiating hemodialysis. In contrast to SMI, SMD showed a negative correlation with BMI, indicating that SMD might reflect dysfunctional sarcopenia better from perspective of the obesity paradox of CKD in this study population.

## Introduction

Sarcopenia is a chronic disease associated with the physiological aging process [1], and chronic kidney disease (CKD) is one of the major factors contributing to its exacerbation [2]. CKD-related factors such as metabolic acidosis, vitamin D deficiency, and inadequate dietary intake have been implicated in the pathogenesis of muscle atrophy [3]. Among patients undergoing dialysis, those with sarcopenia exhibit poorer clinical outcomes compared with their nonsarcopenic counterparts [4]. Accordingly, evaluation in patients with CKD is essential for predicting prognosis and assessing quality of life.

Sarcopenia is characterized by a progressive decline in skeletal muscle mass and strength, which is closely associated with an increased risk of falls and fractures, reduced physical function, and elevated mortality rates [5]. The decrease in skeletal muscle mass is a major characteristic of sarcopenia; however, recent research emphasizes that the skeletal muscle quality may be equally or even more important in determining clinical outcomes [6,7]. The muscle fat infiltration phenomenon, known as myosteatosis, leads to muscle density reduction and muscle quality deterioration and is increasingly being recognized for its significant impact on muscle function and overall health [8–10]. Myosteatosis plays a larger role in muscle tissue dysfunction and increases passive muscle stiffness [11]. Sun et al. demonstrated that computed tomography-defined myosteatosis is independently associated with handgrip strength (HGS) in patients with liver cirrhosis [12]. Wang et al. demonstrated that skeletal muscle density (SMD) derived from a computed tomography (CT) scan strongly associated with higher handgrip strength (HGS) and better performance in the Timed Up and Go test (TUG) in a cross-sectional study conducted on 316 volunteers aged 59–85 years [13]. CT-based low SMD is associated with worsened cardiovascular outcomes and increased mortality across various patient populations–including multiethnicity individuals without pre-existing cardiovascular disease and patients with cardiovascular disease undergoing surgery– highlighting the importance of evaluating both muscle mass and muscle quality in clinical assessments [14,15].

CT is a highly accurate and reliable technique for quantifying skeletal muscle mass and density (measured in Hounsfield units) [16]. CT imaging, particularly at the level of the upper border of the third lumbar vertebra (L3), provides an efficient and representative estimation of the total body muscle mass [17]. CT imaging allows for the body composition analysis using pre-existing scan data and is widely employed as a sarcopenia biomarker in oncology, geriatric, and surgical populations [16,18,19]. In patients with CKD, CT is particularly useful because CT images are less affected by volume overload or edema. Furthermore, CT might also be analyzed for muscle quality by identifying fat infiltration, making it a valuable tool for myosteatosis assessment [16].

This study aims to evaluate the skeletal muscle mass (SMI) and skeletal muscle quality (SMD) using abdominal CT imaging in a homogenous cohort study population as incident hemodialysis patients in single center. Unlike previous studies, such as that by Yajima et al. [20], which included patients undergoing maintenance hemodialysis, our study uniquely focuses on patients at dialysis initiation. This design allows us to assess the clinical significance of the SMI and SMD prior to dialysis-related long-term changes. By retrospectively analyzing the all-cause mortality, we investigate whether low SMI or SMD was associated with poor prognosis. Furthermore, we explore their associations with various nutritional and prognostic clinical parameters to determine which of the two metrics better reflects sarcopenia.

## Materials and methods

### Study population and design

The study was planned as an observational retrospective single-center study for patients with advanced CKD initiating hemodialysis for maintenance. These patients underwent abdominal CT within 1 month around the hemodialysis initiation at Daejeon St. Mary Hospital between January 2018 and December 2023.

A total of 590 patients initiating hemodialysis for maintenance were screened. Patients were insufficient data (*n* = 105) and those who had sufficient data but underwent peritoneal dialysis for maintenance (*n* = 27) patients were excluded. We determined the sex-specific cut-off value for SMD by stratifying the patients by sex. The sex-specific SMD (HU) cut-off values were determined using the Youden index from the receiver operating characteristic (ROC) curve for survival (S1 Fig). The patients were divided into the low- and high-SMD groups for comparison based on the sex-specific cut-off values. They were retrospectively followed until death or censored at the last follow-up, with the observation period ending on October 14, 2024.

This study was approved by the Institutional Review Board of the Catholic Medical Center (DC24RISI0049). This research was presented following the guidelines of the Declaration of Helsinki. All methods were performed according to the relevant guidelines and regulations. The Institutional Review Board of the Catholic Medical Center waived the need for obtaining informed consent because the study was of a retrospective nature.

### Data collection

The baseline characteristics of the demographic and clinical data were adopted by reviewing the electronic medical records including age, sex, height, weight, body mass index (BMI), and comorbidities as causes of CKD (i.e., diabetes, hypertension, and glomerulonephritis).

The laboratory parameters including hemoglobin, differential leukocyte count, albumin, blood urea nitrogen (BUN), serum creatinine, total calcium, corrected calcium, phosphate, uric acid, total carbon dioxide, and intact parathyroid hormone (PTH), and the body measurement data were collected at the closest before the date of hemodialysis commencement. Comorbidity was evaluated using Charlson’s Comorbidity Index (CCI) score with a previously described method [21]. All patients had CKD; hence, a baseline score of 2 was assigned for moderate-to-severe CKD. The data were accessed for research purposes from August 10, 2024, to January 31, 2025, and July 3, 2025, to July 10, 2025. The authors did not have access to any information that could identify individual participants during or after the data collection.

### Calculation of the nutritional markers

As known nutritional markers, geriatric nutritional risk index (GNRI), and the prognostic nutritional index (PNI) score were calculated to assess the nutritional status of the patients. The GNRI and PNI equations are listed below.

The GNRI = [14.89 × serum albumin (g/dL)] + [41.7 × (actual body weight/ideal body weight)], where the ideal body weight was calculated as follows: ideal body weight (kg) = [height (m)]^2^ × 22 (kg/m^2^). The authors set (actual body weight/ideal body weight) as 1 when a patient’s actual body weight was equal to or over the ideal body weight [22]. A GNRI above 98 indicates a normal nutritional status while that between 92 and 98 suggests a mild risk of malnutrition. A GNRI between 82 and 92 suggests a moderate risk, while that under 82 is considered a major risk of malnutrition [22].

The PNI score was calculated as serum albumin (g/L) + 5 x lymphocyte count (10^9^/L) [23]. A PNI score above 45 indicates a good nutritional status, A score of 40–45 suggests mild malnutrition and potential complications. A PNI score below 40 indicates mmoderate-to-severe risk of malnutrition [23].

### Skeletal muscle mass and skeletal muscle density measurements

Abdomen CT scanning was conducted using multidetector row CT (MDCT) scanners, specifically Siemens SOMATOM Definition Flash (dual source 128) and Siemens SOMATOM Force (dual-source 192). The scanning parameters were set at 90 kVp, 109–120 mAs, and a 3 mm slice thickness.

Single-slice transverse nonenhanced CT images at the upper border of the L3 vertebrae were obtained to quantify the skeletal muscle area (SMA, cm^2^), and the SMD (HU).

ImageJ software (version 1.54, NIH, Bethesda, MD, USA) was applied for the Image analysis of obtained single slice of transverse L3 CT. SMA was measured by using an HU threshold range of −29 to +150 for the skeletal muscle, as previously reported [24].

**Fig 1:**
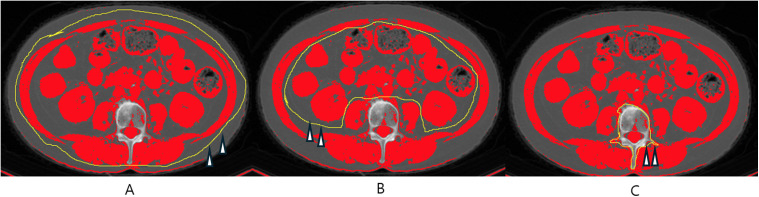
Transverse abdomen computed tomography (CT) images at the level of the third lumbar spine using ImageJ software. The threshold for the skeletal muscle of the CT scan ranges from −29 to + 150 HU. **(A)** Area (cm^2^) of yellow marking (arrowheads) along the outer line of the skeletal muscle mass. **(B)** Area (cm^2^) of yellow marking (arrowheads) along the Inner line of the skeletal muscle mass, **(C)** Area (cm^2^) of yellow marking (arrowheads) along the outer line of L3 vertebral body. The skeletal muscle area (SMA, cm^2^) was obtained by **(A)** –(C). The skeletal muscle density (SMD, HU) measured on SMA, was described as the mean HU using ImageJ software.

With a single-slice transverse L3 CT image, two researchers manually drew the outer and inner musculature perimeters, and the vertebral perimeter in the L3 cross-sectional area using ImageJ software (version 1.54, NIH, Bethesda, MD, USA) (Fig 1). The inner and vertebra perimeter areas were subtracted from the outer perimeter area. The result was obtained as the SMA (cm^2^). The SMI (cm^2^/m^2^) was represented by the SMA corrected by the body height squared. The mean radio density of the obtained SMA at the L3 transverse CT image was described as SMD with the HU using ImageJ software on the abdomen CT scan. [20,25,26]

### Outcome

The primary outcome was all-cause mortality. The SMI and the SMD were evaluated as survival predictors. The patients were divided into categorical low-SMD/high-SMD groups based on Youden’s index of the sex-specific ROC curve for survival. Using the same method, the patients were divided into the low and high SMI groups (S1 Fig). These categorical groups were analyzed for survival prediction, while continuous variables (i.e., SMD and SMI) were evaluated for the same. Subsequently, the correlations between other clinical variables and SMD or SMI were evaluated.

### Statistical analysis

The continuous variables with normal distributions are expressed as mean ± standard deviations, while the categorical variables are indicated as numbers and percentages (%). The continuous variables were compared through a *t*-test. The categorical variables are expressed as numbers with percentages and compared using the chi-squared test. These statistics were used to assess the difference between the low- and high-SMD groups.

SMD and SMI were assessed using the ROC curve for survival to get the optimal cut-off predicting survival. Youden’s index was used to determine the optimal sex-specific cut-off points for SMD and SMI. As continuous variables, SMD and SMI were mainly examined by univariate and multivariate Cox regression models for survival prediction. The Kaplan–Meier survival curves were used to plot the survival curves between groups. The log-rank method was used to determine the statistical significance. The subgroup analysis stratified by the sex and BMI categories for the relationship between SMD and mortality was assessed. The discriminative ability of the Cox proportional hazard models was evaluated by computing Harrell’s concordance index (C-index) using R software (version 4.5.1). The C-indices were compared between the baseline model containing the established risk factors and the extended models, including additional variables (i.e., SMD, CCI, and SMI).

The correlation between the SMD or SMI and the clinical parameters was evaluated using Pearson’s correlation. Multivariable linear regression was performed to verify the independent factors for SMD and SMI in patients initiating hemodialysis. The statistical significance level was set at p < 0.05. Data analyses were performed with SPSS Statistics 27 (IBM Corp., Armonk, NY, USA).

## Result

### Patient characteristics

All 458 patients were enrolled for analysis. Their mean age was 67.8 years, and 300 patients were male (65.5%). Table 1 presents the demographic, clinical, and nutritional characteristics of all patients. As regards comorbidities, 284 (62.0%) patients had diabetes, and 278 (60.7%) patients had hypertension. A total of 204 (44.5%) patients died during the follow-up period.

**Table 1:** The demographic, clinical, and nutritional data of all study patients, and comparisons of Low-SMD and high-SMD groups, stratified by the sex-specific cut-off value drawn with the ROC curves for survival.

	Total	Low SMD	High SMD	p-value
Number of patients	458	264	194	
Age (years)	67.76 ± 14	72.36 ± 12.36	61.50 ± 13.76	< 0.001
Sex (male)	300 (65.5%)	182 (68.9%)	118 (60.82%)	0.071
Laboratory variables				
Hemoglobin (g/dL)	9.17 ± 1.64	9.22 ± 1.67	9.10 ± 1.60	0.459
Creatinine (mg/dL)	7.75 ± 3.71	6.82 ± 3.58	9.04 ± 3.51	< 0.001
BUN (mg/dL)	86.12 ± 30.42	82.48 ± 31.66	91.13 ± 27.97	0.002
Albumin (g/dL)	3.53 ± 0.66	3.38 ± 0.67	3.74 ± 0.59	< 0.001
Total calcium (mg/dL)	8.18 ± 0.96	8.20 ± 0.93	8.16 ± 1.01	0.693
Corrected Calcium (mg/dL)	8.55 ± 0.88	8.69 ± 0.83	8.36 ± 0.92	< 0.001
Intact PTH (pg/mL)	191.47 ± 148.19	158.57 ± 119.53	239.35 ± 166.27	< 0.001
Phosphate (mg/dL)	5.69 ± 1.83	5.35 ± 1.78	6.15 ± 1.80	< 0.001
Uric acid (mg/dL)	7.41 ± 2.72	7.18 ± 2.82	7.71 ± 2.56	0.037
Total CO_2_ (mg/dL)	18.28 ± 4.22	18.35 ± 4.47	18.18 ± 3.89	0.684
Comorbidities				
Diabetes (%)	284 (62.0%)	169 (64.01%)	115 (59.27%)	0.302
Hypertension (%)	279 (60.9%)	160 (60.60%)	119 (61.34%)	0.874
Glomerulonephritis (%)	39 (8.5%)	10 (3.78%)	29 (14.94%)	<0.001
CCI	7.12 ± 2.42	7.82 ± 2.33	6.14 ± 2.20	<0.001
Body composition				
BMI (kg/m^2^)	24.08 ± 4.29	24.52 ± 4.62	23.48 ± 3.73	0.008
Height (cm)	162.08 ± 9.05	161.62 ± 8.92	162.71 ± 9.21	0.202
Weight (kg)	63.5 ± 13.98	64.43 ± 14.86	62.46 ± 12.63	0.127
Waist (mm)	943.01 ± 120.39	968.00 ± 122.12	909.00 ± 109.44	<0.001
SMA (cm^2^)	125.81 ± 36.65	127.43 ± 39.72	123.63 ± 31.98	0.259
SMI (cm^2^/m^2^)	47.53 ± 11.43	48.30 ± 12.56	46.48 ± 9.62	0.080
Nutrition				
GNRI	99.26 ± 13.08	97.82 ± 13.69	101.22 ± 11.95	0.006
PNI	40.96 ± 8.07	39.17 ± 8.33	43.40 ± 7.03	<0.001

HD, hemodialysis; BUN, Blood urea nitrogen; iPTH, intact parathyroid hormone; CCI, Charlson Comorbidity Index; SMA, skeletal muscle area; SMI, skeletal muscle index; GNRI, geriatric nutritional risk index; and PNI, prognostic nutrition index.

The SMD cut-off values from the ROC curves for mortality prediction by Youden’s index were 21.75 and 33.55 HU for women and men, respectively (S1 Fig). The study population was divided into the low- and high-SMD groups based on the sex-specific cut-off values. The demographic, clinical, and nutritional characteristics of the two groups were compared. The low-SMD group was significantly older and more obese and had a higher number of death. They also showed lower serum creatinine, lower albumin, lower intact PTH, higher corrected calcium, lower phosphate, higher waist circumference, higher CCI score, more emergency hemodialysis, and poorer nutritional states.

### Survival curve of the low-SMD group vs the high-SMD group

**Fig 2:**
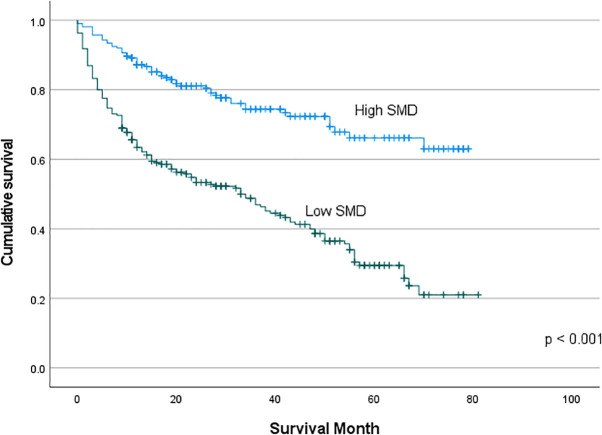
Kaplan–Meier analyses of the survival for all-cause mortality comparisons of the low-SMD group versus the high-SMD group based on the L3 level abdominal CT image.

Using Youden’s index in the sex-specific ROC curve for survival, the best cut-off values of SMD for predicting mortality were 21.75 HU for women and 33.55 HU for men. The cut-off value of the SMI for predicting mortality in women was 29.46 cm^2^/m^2^, while that for men was 43.53 cm^2^/m^2^ (S1 Fig). The cumulative survival of the low-SMD group versus the high-SMD group was compared by using Kaplan-Meier survival curves (Fig 2). The cumulative survival of the low-SMD patients was significantly worse than that of the high-SMD ones (p < 0.001). The low- versus high-SMI group comparison on Kaplan-Meier survival curves were then analyzed. The low-SMI group showed worse survival compared to the high-SMI group (S2 Fig).

### Prognostic factors for survival

Table 2 presents the univariate Cox proportional hazard regression models for the overall survival of the study populations. In the univariate analysis, older age, higher Charlson comorbidity score, emergency hemodialysis, shorter height, lower BMI, lower SMA, lower SMI, lower SMD, low SMI (categorical), low SMD (categorical), lower serum creatinine, lower albumin, higher corrected calcium, lower GNRI and lower PNI were statistically significant risk factors of all-cause mortality in this study population. Using a sex-specific cut-off SMD value determined by the ROC curves, the low-SMD (categorical) showed the highest HR (2.963, 95% CI 2.170–4.047, *p* < 0.001) for predicting the mortality.

**Table 2:** Univariate Cox proportional hazards analysis of all-cause death in all study populations.

Variable	Univariate analysis		
	Hazard ratio	95% Confidence interval	*p*-value
Age	1.060	1.048–1.073	<0.001
Male sex	0.767	0.580–1.015	0.064
Emergency HD	2.362	1.658–3.365	<0.001
Comorbidities			
Hypertension	0.875	0.662–1.156	0.347
Diabetes	0.927	0.699–1.228	0.596
CCI	1.266	1.198–1.338	<0.001
Body composition			
Weight (kg)	0.979	0.968–0.989	<0.001
Height (cm)	0.973	0.958–0.987	<0.001
BMI (kg/m^2^)	0.956	0.923–0.989	0.011
Waist (mm)	1.000	0.999–1.001	0.699
SMI (cm^2^/m^2^)	0.979	0.966–0.992	0.002
SMA (cm^2^)	0.991	0.987–0.996	<0.001
SMD (HU)	0.938	0.924–0.951	<0.001
Low SMD	2.963	2.170–4.047	< 0.001
Low SMI	2.224	1.633–3.028	< 0.001
Laboratory variable			
Hemoglobin	0.979	0.897–1.068	0.631
BUN	0.993	0.988–0.998	0.004
Creatinine	0.837	0.798–0.879	<0.001
Albumin	0.616	0.502–0.756	<0.001
Intact PTH	0.999	0.998–1.000	0.033
Total Calcium	1.019	0.883–1.176	0.797
Corrected calcium	1.306	1.106–1.542	0.002
Phosphate	0.862	0.790–0.939	<0.001
Total CO2	1.020	0.985–1.057	0.267
Uric acid	0.989	0.938–1.042	0.669
Nutrition			
GNRI	0.972	0.962–0.983	<0.001
PNI	0.954	0.937–0.970	<0.001

Low SMD: men SMD ≤ 33.55 HU, women SMD ≤ 21.75 HU; Low SMI: men SMI ≤ 43.53 cm^2^/m^2^, women ≤ 29.46 cm^2^/m^2^; HD: hemodialysis, and CCI: Charlson Comorbidity Index.

Table 3 shows the multivariate Cox proportional hazard regression analysis results for SMD, SMI, and low SMD. The univariate analysis results were presented separately for comparison. In the multivariate analysis adjusted for age, sex, BMI, albumin, phosphate, and corrected calcium, SMD was associated with a reduced hazard ratio of 0.961 and remained statistically significant. After a multivariate analysis adjustment, the SMI was found to be insignificant in the reduction of the hazard ratio for mortality. The low-SMD group (below sex-specific cut-off values of SMD) remained statistically significant as an independent risk factor for mortality, showing a 1.805 hazard ratio after adjustment in the multivariate analysis.

**Table 3:** Univariate and multivariate Cox regression analyses of SMD, SMI, and low-SMD.

Model	Variable	Hazard ratio (95% Confidence interval)	*p*-value
Univariate	SMD (per unit increase)	0.938 (0.924–0.951)	< 0.001
	SMI (per unit increase)	0.979 (0.966–0.992)	0.002
	Low-SMD (vs. high SMD)	2.963 (2.170–4.047)	< 0.001
Multivariate	SMD (per unit increase)	0.961 (0.942–0.980)	< 0.001
	SMI (per unit increase)	1.002 (0.986–1.019)	0.778
	Low-SMD (vs. high SMD)	1.805 (1.263–2.579)	0.001

Multivariate Cox proportional hazard model adjusted for age, sex, BMI, albumin, phosphate, and corrected calcium levels. Low SMD; SMD ≤ 21.75 HU for women or SMD ≤ 33.55 HU for men.

### Subgroup analysis of the HR of SMD for all-cause mortality

Subgroup analyses were performed according to the sex and BMI categories (BMI < 25 and BMI ≥ 25), given their potential modifying effect on the SMD and mortality association. Multivariate Cox proportional hazard models were used in each subgroup after adjusting for age, sex, BMI, albumin, phosphate, and corrected calcium levels. In both men and women, SMD was associated with a significant risk ratio reduction. In both the BMI subgroups (BMI < 25 and BMI ≥ 25), SMD showed a significant risk ratio reduction. The subgroup analysis showed that SMD was associated with a significant risk reduction, regardless of the sex and BMI (Table 4).

**Table 4:** Subgroup analysis according to sex and BMI of SMD and mortality using multivariate Cox proportional hazard models adjusted for age, sex, BMI, albumin, phosphate, and corrected calcium.

Subgroup	N	Hazard ratio (95% Confidential interval)	*p-*value for the interaction
Sex			0.906
Men	300	0.963 (0.939–0.988)	
Women	158	0.956 (0.926–0.988)	
BMI			0.666
BMI < 25	280	0.965 (0.942–0.988)	
BMI ≥ 25	178	0.955 (0.919–0.992)	

### Model discrimination in predicting all-cause mortality

Harrell’s C-index was used to evaluate the discriminative ability of the Cox proportional hazard model in Table 5 and consequently validate SMD compared with the CCI and SMI in predicting all-cause mortality.

**Table 5:** Predictive accuracies of SMD compared with the CCI and SMI for all-cause mortality.

Variable	C-index	Standard error
Baseline model	0.732 (0.697–0.767)	0.018
+ SMD	0.747 (0.714–0.780)	0.017
+ CCI	0.737 (0.702–0.772	0.018
+ SMI	0.732 (0.697–0.767)	0.018

Baseline model: Cox proportional hazard analysis model adjusted for age, sex, BMI, albumin, corrected calcium, and phosphate; SMD: skeletal muscle density; CCI: Charlson Comorbidity Index; SMI: skeletal muscle index.

The baseline model is a multivariate Cox proportional hazard model adjusted for age, sex, BMI, albumin, corrected calcium, and phosphate. Adding SMD to the baseline model significantly improved the C-index from 0.732 to 0.747. SMD improved the C-index of the baseline model more than the CCI dose. Meanwhile, the SMI did not improve the baseline model showing the same C-index as the baseline model.

### Clinical variables correlated with SMD or SMI

A regression analysis was performed to investigate the impact of the clinical variables on SMD or SMI. In the univariate regression analysis, SMD showed a significant positive correlation with male sex (*r* = 0.398, *p* < 0.001), height (*r* = 0.383, *p* < 0.001), SMA (*r* = 0.171, *p* = 0.001), albumin (*r* = 0.321, *p* < 0.001), phosphate (*r* = 0.180, *p* < 0.001), BUN (*r* = 0.225, *p* < 0.001), creatinine (*r* = 0.389, *p* < 0.001), intact PTH (*r* = 0.183, *p* < 0.001), GNRI (*r* = 0.138, *p* = 0.003) and PNI (r = 0.318, *p* < 0.001). SMD showed a significant negative correlation with age (r = −0.465, p < 0.001), BMI (*r* = −0.124, *p* = 0.008), perimeter (*r* = −0.206, *p* < 0.001), corrected Ca (*r* = −0.190, *p* < 0.001), and CCI (r = −0.358, *p* < 0.001). A multivariable linear regression analysis was subsequently performed, focusing on factors that were significant in the simple regression analysis. Importantly, the multivariable linear regression analysis revealed that male sex (β = 0.340, *p* < 0.001), creatinine (β = 0.133, p < 0.001) and albumin (β = 0.242, p < 0.001) remained significant and had an independently positive correlation with SMD, while age (β = −0.366, *p* < 0.001), and BMI (β = −0.199, p < 0.001) remained significant and had an independent negative correlation with SMD in Table 6.

**Table 6:** Regression analysis of the associations between the L3 SMD and the clinical variables. In the univariate and multivariate adjustment.

Variable	Univariate analysis		Multivariate analysis	
	R	*p*-value	beta	*p* -value
Gender male	0.398	<0.001	0.340	<0.001
Age	−0.465	<0.001	−0.366	<0.001
Height	0.383	<0.001		
Weight	0.087	0.063		
BMI, kg/m^2^	−0.124	0.008	−0.199	<0.001
SMI	0.051	0.275		
SMA	0.171	0.001		
Perimeter	−0.206	<0.001		
Albumin	0.321	<0.001	0.242	<0.001
Corrected Ca, mg/dL	−0.190	<0.001		
Phosphate	0.180	<0.001		
BUN	0.225	<0.001		
Creatinine, mg/dL	0.389	<0.001	0.133	<0.001
Hemoglobin, g/dL	0.014	0.766		
Intact PTH	0.183	<0.001		
TCO2	0.022	0.656		
Uric acid	0.107	0.023		
GNRI	0.138	0.003		
PNI	0.318	<0.001		
CCI	−0.358	<0.001		

BMI: body mass index; SMI: skeletal mass index; SMA: skeletal mass area; and CCI: Charlson Comorbidity Index.

In the univariate analysis of the SMI, the SMI showed a significant positive correlation with male sex (*r* = 0.295, *p* < 0.001), BMI (*r* = 0.520, *p* < 0.001) height (*r* = 0.260, *p* < 0.001), weight (*r* = 0.564, *p* < 0.001), SMA(*r* = 0.918, *p* < 0.001), perimeter (*r* = 0.490, *p* < 0.001), creatinine (*r* = 0.194, *p* < 0.001) and GNRI (r = 0.253, *p* < 0.001). The SMI showed a significant negative correlation with age (*r* = −0.236, *p* < 0.001) and corrected calcium (*r* = −0.205, *p* < 0.001). A multivariable linear regression analysis was conducted, focusing on the significant factor in the simple regression analysis. The male sex (*β* = 0.244, *p* < 0.001) and BMI (*β* = 0.485, *p* < 0.001) remained significant and had an independently positive correlation with the SMI, while age (*β* = −0.125, *p* < 0.001) and corrected calcium (*β* = −0.133, *p* < 0.001) remained significantly and showed an independently negative correlation with the SMI in S1 Table. The BMI represents a point of contrast in the respective significant negative correlation with the SMD and positive correlation with the SMI.

## Discussion

The low SMD below sex-specific cut-off value of 21.75 HU for women or 33.55 HU for men and the lower SMD measured by L3 abdominal CT scan were significant risk factors predicting the survival of patients with advanced CKD initiating hemodialysis.

Research on sarcopenia in patients with CKD using imaging has considered skeletal muscle mass as a sarcopenia marker early on. Sabatino et al. showed low SMA in the L3 level assessed by abdominal CT, as representative of skeletal muscle mass, was independently associated with all-cause mortality in patients with end-stage kidney disease on hemodialysis [27]. Yajima et al. assessed psoas muscle thickness per height in patients with hemodialysis and verified that a lower psoas muscle thickness is associated with mortality. [28] Recent research on sarcopenia by image study has emphasized the quality of skeletal muscle, and a few studies have assessed SMD in patients with CKD. In the work by Donato et al., 167 Portuguese nondialysis patients underwent the L3 CT scan, showing that a lower SMD can predict increased mortality [29]. Kim et al. studied nondialysis CKD patients and verified that CKD progression and mortality were associated with myosteatosis [30]. Meanwhile, as a form of a short report, Yajima et al. demonstrated that the SMD is independently associated with all-cause mortality in Japanese 242 patients on hemodialysis already for more than 6 months. In our study, we investigated the association between SMI- and SMD-based L3 abdominal CT images and subsequent mortality in 458 patients on incident hemodialysis. To the best of our knowledge, this is the first study to elucidate the association between sarcopenia as the SMI and SMD by an L3 abdominal CT image and mortality in a homogenous cohort of patients on incident hemodialysis. We have verified that the SMI and SMD based on an L3 abdominal CT image were strong predictors of mortality in patients with advanced CKD initiating hemodialysis, provided detailed numerical data and clinically meaningful results.

In our study, the low SMD group, which was defined to have SMD ≤ 21.75 HU for women or SMD ≤ 33.55 HU for men, as sex-specific cut-off values based on the Youden index derived from the ROC curve, showed a significant hazard ratio of 1.805 [95% CI 1.263–2.579] for mortality in the Cox proportional hazards analysis in multivariate adjustments. The continuous SMD remained significant in risk reduction as 0.961 [95% CI 0.942–0.980] for mortality in this study population. Compared with the SMI in terms of prognostic power for predicting mortality, the SMI lost its power as a prognostic marker after adjustment in the multivariate Cox proportional hazard analysis. Similar findings have been reported in the general population, showing that SMD predicts mortality better than the SMA or SMI. In the multiethnicity prospective cohort study with 1,974 participants using the CT scan, the greater muscle density, not the muscle area, was associated with a markedly lower risk of all-cause mortality over 11 years of follow-up [15]. A total of 8,303 patients undergoing routine colon cancer screening CT showed that myosteatosis increases the hazard ratio by 1.89 [95% CI 1.52–2.35] at *p* < 0.001 after adjustment [8]. SMD itself has a strong prognostic value. In the subgroup analyses stratified by sex and BMI, SMD was consistently associated with reduced risk of mortality across all subgroups. We used Harrell’s C-index to evaluate the discriminative power of the Cox proportional hazard model. Adding SMD to the baseline model (age, sex, BMI, albumin, phosphate, and corrected calcium) improved the C-index even more than the Charlson comorbidity score.

The SMD validates skeletal muscle fat infiltration and is affected by several clinical variables. Avesani et al. stated that skeletal muscle fat infiltration is a product of sex hormones, aging, physical inactivity, and obesity [31]. In this study population, SMD was positively correlated with male sex, albumin, and creatinine levels but negatively correlated with age and BMI. In this population, younger age, male sex, and higher BMI were correlated with increased SMI, and this correlation was established in a previous study [32–33]. Age showed a strong correlation with SMD, as many studies have reported [34–36]. Interestingly, SMD in this population showed a negative correlation with the BMI (β = −0.199, p < 0.001), while SMI depicted a strong positive correlation (β = 0.485, p < 0.001). Even if the BMI included muscle mass as well, it increased with obesity. A BMI above 25 in an Asian population may be associated with fat tissue infiltration in the muscle layer [37]. In patients with advanced CKD, the BMI has limitations in assessing the status of volume overload or myosteatosis status. Compared to the SMI, the SMD reflects more sarcopenia dissociated with obesity in patients with advanced CKD.

Another strong point of this study is that serum albumin also showed a positive correlation with the SMD (*β* = 0.242, *p* < 0.001), but not with the SMI. The similar result that albumin did not show a correlation with the muscle area was reported on research targeted on healthy kidney donor CT analysis [33]. A recent study demonstrated that albumin can be reverse relation with acute inflammation [38]. The decreasing SMD is associated with insulin resistance and chronic inflammation [39]. The ectopic fat depot in skeletal muscle visceral fat may release a proinflammatory cytokine, resulting in local inflammation [39,40]. Sach et al. demonstrated that intermuscular adipose tissue (IMAT) may promote both skeletal muscle insulin resistance and secretion of inflammatory cytokines by incubating human primary muscle cells in conditioned media from IMAT. RNA sequencing was performed on IMAT, as well [41]. Accordingly, IMAT secretes factors that modulate muscle insulin sensitivity via secretion of inflammatory cytokines and extracellular matrix proteins [41]. In this aspect the considering the association with the albumin level, the SMD showed a more significant relation with the inflammation than the SMI. The CCI, which represents the weighted comorbid condition, showed a strong negative correlation with the SMD (*r* = −0.358, *p* < 0.001) but not with the SMI (*r* = −0.072, *p* = 0.125). The SMD was more closely associated with the comorbid conditions, but the SMI was not. This may be the reason why the SMD has more predictive power in mortality than the SMI.

Additional mechanisms linking low SMD to increased mortality is impaired insulin sensitivity and atherosclerosis development. Inflammation plays a central role in exacerbating both insulin resistance and atherosclerosis. Ectopic fat accumulation in the skeletal muscle—reflected by low SMD—has been identified as a key contributor to insulin resistance and atherosclerotic changes in several studies [42,43]. A large cross-sectional study conducted in Korea, including 18,251 participants, demonstrated that a higher homeostatic model assessment for insulin resistance was significantly associated with an increased risk of low SMD [42]. Furthermore, myosteatosis is a risk factor for coronary artery calcification in patients with type 2 diabetes [43]. Both insulin resistance and atherosclerosis are closely linked to cardiovascular mortality; hence, these findings support the prognostic value of the low SMD.

This study has some limitations. First, owing to the retrospective nature of this study, the authors cannot exclude that residual confounding may exist. Second, the L3SMI and L3SMD values used for the data analyses were measured only at the initiation of hemodialysis, and any changes in these values during the follow-up period were not evaluated. Third, the study group was confined to patients with advanced CKD initiating hemodialysis, hence, no control or reference ranges of the SMD and SMI existed. Fourth, two researchers measured the SMD and the SMI manually drawing the outer and inner musculature and vertebra perimeter, and observer bias may exist.

## Conclusions

This study provides strong evidence that SMD, a marker of muscle quality, is an independent and powerful predictor of mortality in patients with advanced CKD initiating hemodialysis. Compared with the SMI that reflects muscle quantity, SMD offers superior prognostic value, likely due to its association with inflammation, metabolic dysfunction, and muscle fat infiltration. Future prospective studies are warranted to confirm these results and explore therapeutic strategies aimed at improving muscle density and overall clinical outcomes in patients undergoing dialysis.

## Supporting information

S1 Fig Sex-stratified AuROC analysis was used to assess the predictive power of SMI and SMD for mortality. (A) Women, (B) Men. AuROC; Area under the receiver operating characteristic curve, SMI; Skeletal muscle index, SMD; Skeletal muscle density.
(TIF)

S2 Fig Kaplan-Meier analyses are performed to examine the association between L3 CT scan muscle index and all-cause mortality. (TIF)

S1 Table Regression analysis of the associations between SMI and baseline variables. (DOCX)

S1 File Patients’ raw data. (XLSX)
