# Supplementary figures and images for: Abdominal computed tomography–assessed muscle quality and its prognostic value in patients with advanced chronic kidney disease initiating hemodialysis
Source: PLoS One. 2025 Nov 4;20(11):e0334929. doi: 10.1371/journal.pone.0334929 (PMC12585029; doi:10.1371/journal.pone.0334929)

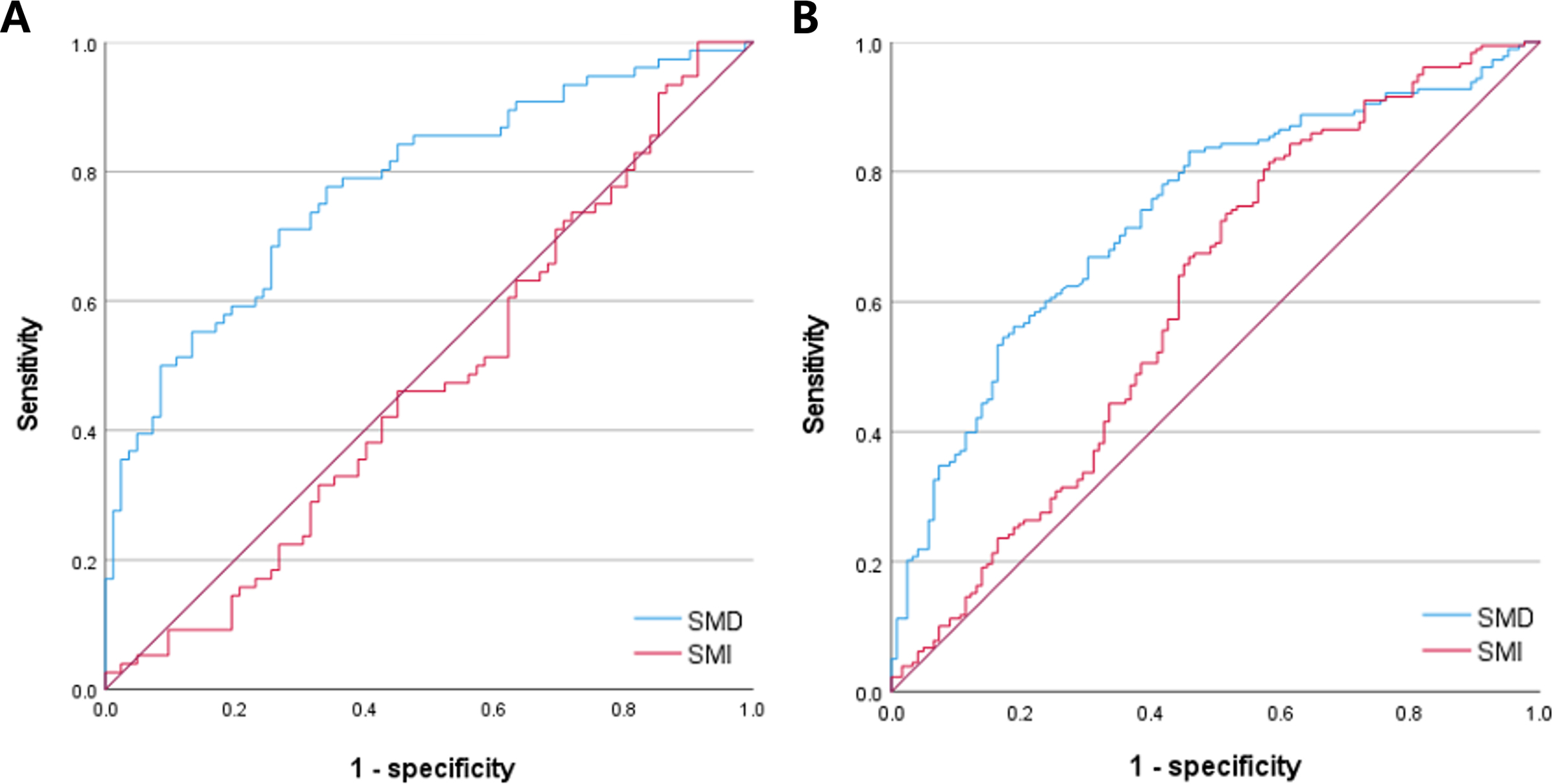

Supplement: S1 Fig — (A) Women, (B) Men. AuROC; Area under the receiver operating characteristic curve, SMI; Skeletal muscle index, SMD; Skeletal muscle density. (TIF) [file pone.0334929.s001.tif]

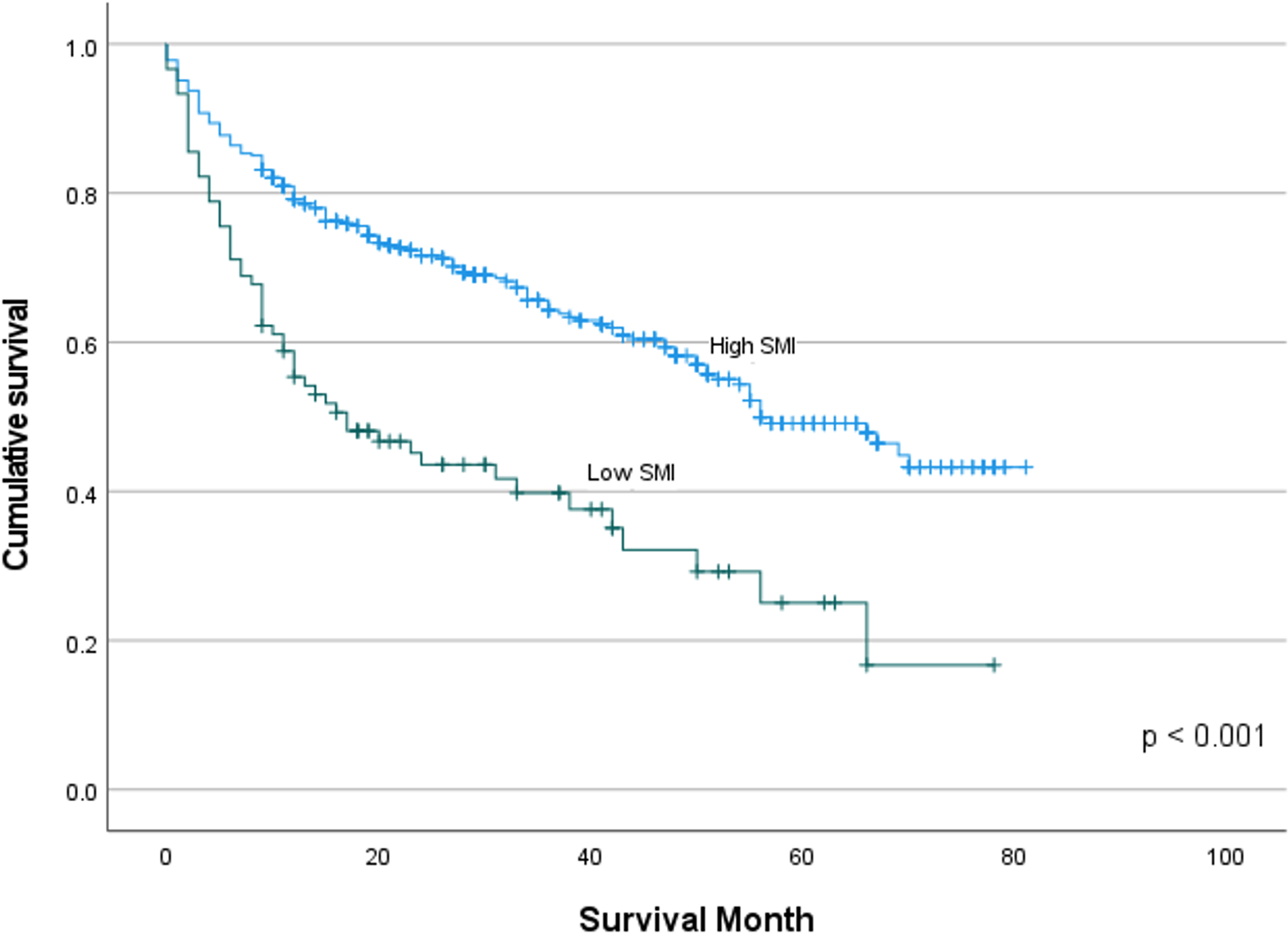

Supplement: S2 Fig — (TIF) [file pone.0334929.s002.tif]
