# Supplementary material for: Abdominal computed tomography–assessed muscle quality and its prognostic value in patients with advanced chronic kidney disease initiating hemodialysis
Source: PLoS One. 2025 Nov 4;20(11):e0334929. doi: 10.1371/journal.pone.0334929 (PMC12585029; doi:10.1371/journal.pone.0334929)
Supplement: S1 Table — (DOCX) [file pone.0334929.s003.docx]

Table S1

| Variable | Univariate analysis | | Multivariate analysis | |
| --- | --- | --- | --- | --- |
|  | R | p value | beta | p value |
| Gender male | 0.295 | <0.001 | 0.244 | <0.001 |
| Age | −0.236 | <0.001 | −0.125 | <0.001 |
| Height | 0.260 | <0.001 |  |  |
| Weight | 0.564 | <0.001 |  |  |
| BMI, kg/m2 | 0.520 | <0.001 | 0.485 | <0.001 |
| SMD | 0.051 | 0.275 |  |  |
| SMA | 0.918 | <0.001 |  |  |
| Perimeter | 0.490 | <0.001 |  |  |
| Albumin | −0.079 | 0.091 |  |  |
| Ca | −0.231 | <0.001 |  |  |
| Corrected Ca, mg/dL | −0.205 | <0.001 | −0.133 | <0.001 |
| P | 0.077 | 0.101 |  |  |
| BUN | 0.000 | 0.992 |  |  |
| Creatinine, mg/dL | 0.194 | <0.001 |  |  |
| Hemoglobin, g/dL | −0.079 | 0.093 |  |  |
| Intact PTH | −0.025 | 0.609 |  |  |
| TCO2 | 0.013 | 0.783 |  |  |
| Uric acid | −0.043 | 0.366 |  |  |
| GNRI | 0.253 | <0.001 |  |  |
| PNI | −0.048 | 0.310 |  |  |
| CCI | −0.072 | 0.125 |  |  |
